# How smartphone usage correlates with social anxiety and loneliness

**DOI:** 10.7717/peerj.2197

**Published:** 2016-07-12

**Authors:** Yusong Gao, Ang Li, Tingshao Zhu, Xiaoqian Liu, Xingyun Liu

**Affiliations:** 1 School of Computer and Control, University of Chinese Academy of Sciences, Beijing, China; 2 Key Laboratory of Behavioral Science, Institute of Psychology, Chinese Academy of Sciences, Beijing, China; 3 Department of Psychology, Beijing Forestry University, Beijing, China

**Keywords:** Smartphone usage, Loneliness, Social anxiety

## Abstract

**Introduction:** Early detection of social anxiety and loneliness might be useful to prevent substantial impairment in personal relationships. Understanding the way people use smartphones can be beneficial for implementing an early detection of social anxiety and loneliness. This paper examines different types of smartphone usage and their relationships with people with different individual levels of social anxiety or loneliness.

**Methods:** A total of 127 Android smartphone volunteers participated in this study, all of which have agreed to install an application (MobileSens) on their smartphones, which can record user’s smartphone usage behaviors and upload the data into the server. They were instructed to complete an online survey, including the Interaction Anxiousness Scale (IAS) and the University of California Los Angeles Loneliness Scale (UCLA-LS). We then separated participants into three groups (high, middle and low) based on their scores of IAS and UCLA-LS, respectively. Finally, we acquired digital records of smartphone usage from MobileSens and examined the differences in 105 types of smartphone usage behaviors between high-score and low-score group of IAS/UCLA-LS.

**Results:** Individuals with different scores on social anxiety or loneliness might use smartphones in different ways. For social anxiety, compared with users in low-score group, users in high-score group had less number of phone calls (incoming and outgoing) (*Mann-Whitney U* = 282.50∼409.00, *p* < 0.05), sent and received less number of text messages in the afternoon (*Mann-Whitney U* = 391.50∼411.50, *p* < 0.05), used health & fitness apps more frequently (*Mann-Whitney U* = 493.00, *p* < 0.05) and used camera apps less frequently (*Mann-Whitney U* = 472.00, *p* < 0.05). For loneliness, users in low-score group, users in high-score group had less number of phone calls (incoming and outgoing) (*Mann-Whitney U* = 305.00∼407.50, *p* < 0.05) and used following apps more frequently: health & fitness (*Mann-Whitney U* = 510.00, *p* < 0.05), system (*Mann-Whitney U* = 314.00, *p* < 0.01), phone beautify (*Mann-Whitney U* = 385.00, *p* < 0.05), web browser (*Mann-Whitney U* = 416.00, *p* < 0.05) and social media (RenRen) (*Mann-Whitney >U* = 388.50, *p* < 0.01).

**Discussion:** The results show that individuals with social anxiety or loneliness receive less incoming calls and use healthy applications more frequently, but they do not show differences in outgoing-call-related features. Individuals with higher levels of social anxiety also receive less SMSs and use camera apps less frequently, while lonely individuals tend to use system, beautify, browser and social media (RenRen) apps more frequently.

**Conclusion:** This paper finds that there exists certain correlation among smartphone usage and social anxiety and loneliness. The result may be useful to improve social interaction for those who lack social interaction in daily lives and may be insightful for recognizing individual levels of social anxiety and loneliness through smartphone usage behaviors.

## Introduction

The quality of personal relationships has an enormous impact on our physical and psychological health. It indicates that factors that inhibit interpersonal functioning need to be investigated. Within the field of psychology, both social anxiety and loneliness are important factors contributing to poor-quality relationships (Peplau & Perlman, 1982; García-López et al., 2008). Individual experience of loneliness and social anxiety can be hindered in building their social connections. Specifically, social anxiety refers to “anxiety resulting from the prospect or presence of personal evaluation in real or imagined social situations,” while, loneliness refers to “the experience of emotional and social isolation” (Schlenker & Leary, 1982). Early detection of social anxiety and loneliness might be useful to prevent substantial impairment in personal relationships (Bokhorst, Goossens & de Ruyter, 2001; Drageset et al., 2015). However, it is very difficult for traditional methods (e.g. face-to-face survey or interview) to track the changes of an individual’s social anxiety and loneliness over time.

The emergence of smartphones may shed some light on this direction. Currently, smartphones have become increasingly popular around the world, and have become a necessity for individuals in modern times. According to the International Data Corporation (IDC) Worldwide Quarterly Mobile Phone Tracker, in 2014, worldwide smartphone shipments reached a total of 1.3 billion units (Llamas, 2015). In addition to basic cellphone capabilities (e.g. voice calling and text messaging), the smartphone is built with more convenient features that facilitate communication like a computer. Users can download applications from digital distribution platforms (e.g. Google Play and App Store) to expand their smartphone functionality (e.g. social communication, entertainment, and Internet surfing). More importantly, digital records of individual’s smartphone usage data can be collected and processed in a real-time, continuous, and non-intrusive manner.

Smartphone usage can provide behavioral cues to individual’s psychological features. Early studies found that relationships exist between mobile phone use behaviors and psychological features (e.g. personality, self-esteem, impulsivity, and well-being) (Ehrenberg et al., 2008; Billieux, Van der Linden & Rochat, 2008; Gross, Juvonen & Gable, 2002; Butt & Phillips, 2008). A few recent studies explored this further. Chittaranjan, Blom & Gatica-Perez (2011) found that individual’s Big-Five personality traits can be manifested on their smartphone usage behaviors. Montag et al. (2014) collected digital records of individual’s smartphone usage data via an Android application (Menthal), and found that a relationship exists between an individual’s personality and digital records of smartphone usage. LiKamWa et al. (2013) collected digital records of smartphone usage data, and found that digital records of smartphone usage data indicate changes in emotions. Previous studies suggest that individual’s psychological features can be identified through their smartphone usage behaviors. However, Asselbergs et al. (2016) replicated this study and did not get so positive findings, and they recommended that more advanced data mining techniques should be developed in future studies. Understanding the way targeted persons (e.g. individuals with higher levels of social anxiety and loneliness) use smartphones can be helpful for identifying those people among populations at an early stage. However, there are few studies done to examine the relationship between smartphone usage behaviors and social anxiety or loneliness.

This correlational study aims to examine the relationship between digital records of smartphone usage data and social anxiety or loneliness, and investigate differences in smartphone usage behaviors among users with different levels of social anxiety and loneliness.

## Methods

The procedure of our work consists of 2 steps: (1) **Data collection** and (2) **Data analysis**. Methods and procedures of this study were approved by the Institutional Review Board of the Institute of Psychology, Chinese Academy of Sciences, H09036.

### Data collection

**Figure 1:**
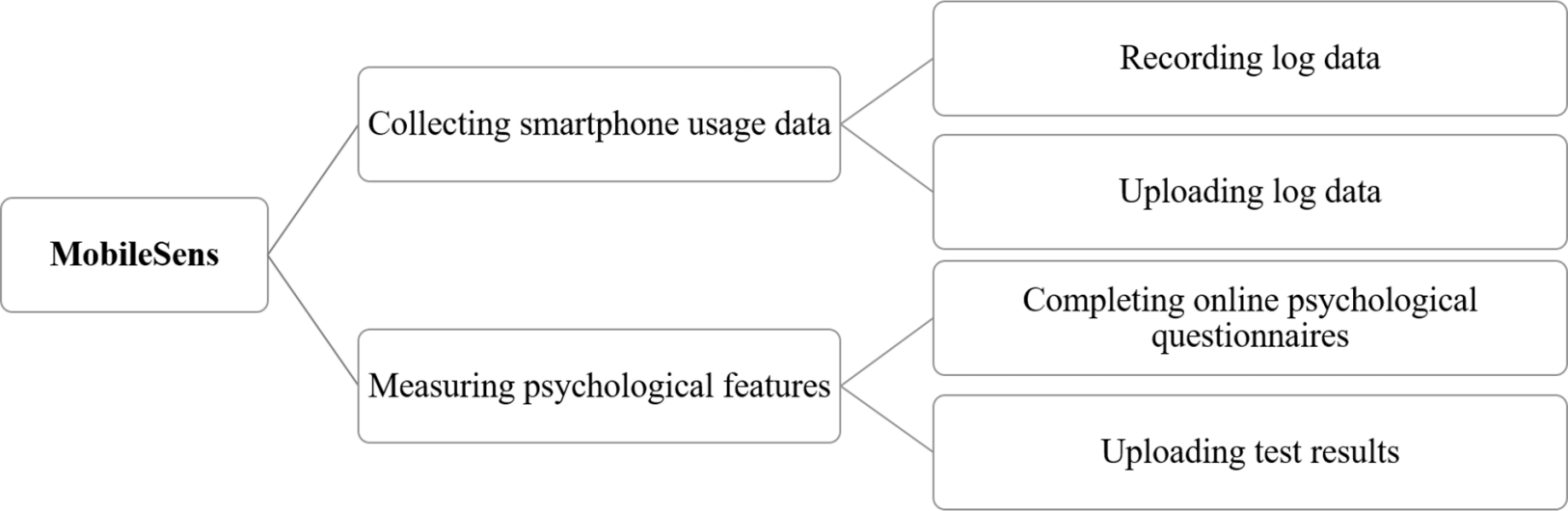
Outline of MobileSens.

We broadcasted participant invitation on Chinese social networking websites (Sina Weibo and RenRen) in May 2013, and obtained electronic informed consent. Participants were expected to accept our invitation before June 2013. Participants can be selected according to the following criteria:

- Since we used an Android application (MobileSens) to collect data (see Fig. 1) (Guo et al., 2011; Li et al., 2013) for this study, all participants should be Android smartphone users.
- Because new users may use their phones in an irregular manner (such as installing a large number of apps or creating a large number of new contacts), participants should also have been using their smartphones for more than three months.

Due to limited resources (e.g. time, manpower, and server resources), we only invited 150 participants, and finally a total of 146 qualified participants agreed to participate in this study.

During this study, all participants were instructed to install MobileSens on their smartphones. To obtain enough smartphone usage data for further analysis, participants were required to use MobileSens for more than 30 days. Once the study was done, we reminded participants to complete online psychological questionnaires via their smartphones. Once participants finished uploading 30 days data and completed the questionnaires, we rewarded them with 200 RMB and sent them detailed instruction to uninstall MobileSens. However, if participants dropped out of the study after finishing questionnaires and uploading data for less than 30 days, we calculated their number of days uploading data after the experiment and gave them parts of the experiment reward.

#### Smartphone usage data

We collected individual’s smartphone usage data via MobileSens. Once users installed MobileSens on their Android smartphones, it ran as a backend service to record different types of smartphone usage data, excluding private information such as actual content of voice calls or text messages (see Table 1), and uploaded collected data to the server.

**Table 1:** Details of smartphone log data.

Primary category	Definition
Activity application log	Creating, starting, resuming, stopping, and exiting of different **activities in applications**
Application package log	Adding, changing, and removing package
Calling log	State, number, contact, and direction of calling
Configuration log	Configuration change information (e.g., font, screen size, and keyboard type)
Contact log	Adding, changing, and deleting of contacts
Date changed log	Changing of system date and time
GPS log	User’s locale, altitude, latitude, longitude and direction of movement
Headset log	Plugging in headset or not
Power connected log	Connecting or disconnecting the power
Power log	Powering on smartphone or not
Screen log	State of the screen (ON/OFF)
Service application log	Creating, starting, and deleting service application
SMS log	State, and contacts of SMS
Wallpaper log	Changing wallpaper

#### Questionnaires

The online survey model in MobileSens applied questionnaires consisting of some basic demographic questions (including gender and age), Interaction Anxiety Scale (IAS) and UCLA Loneliness Scale (UCLA-LS).

IAS is an effective tool designed to measure social anxiety (Leary & Kowalski, 1993). It consists of 15 self-rating items. Participants rated themselves on each item by a 5-point Likert Scale. High scores indicate high levels of social anxiety. While, the UCLA-LS is a self-report measure of loneliness (Russell, 1996). The UCLA-LS is a 4-point Likert Scale, consisting of 20 self-rating items. High scores indicate high levels of loneliness.

All participants were required to complete all the online questionnaires (IAS and UCLA-LS) via their smartphones and uploaded data to the server.

After data collection, we excluded a portion of participants based on the following criteria: (a) participants who were less than 18 years old; (b) participants who provided invalid answers on the online questionnaires (identified by polygraph questions and too short item filling time); (c) participants who did not upload enough smartphone data to the server. Finally, we acquired data from a total of 127 participants (23.66 ± 2.86 years old; men: 74; women: 53).

### Data analysis

#### Smartphone usage behaviors

After collecting raw data (smartphone usage log data), we created variables for further analysis. In this study, we created 105 types of smartphone usage behaviors by following three steps:

- We designed some basic behaviors (e.g. frequency of using text messages, making phone calls, changing wallpaper, switching on screen, and playing game) based on previous research investigating smartphone usage behaviors (Butt & Phillips, 2008; Reid & Reid, 2007; Thomée, Härenstam & Hagberg, 2011; Güzeller & Coşguner, 2012; Gao, Li & Zhu, 2014; Gao, Lei & Zhu, 2015; LiKamWa et al., 2013; Chittaranjan, Blom & Gatica-Perez, 2011).
- We examined individual’s preference for using different types of apps and games. Based on a classification framework (https://www.wandoujia.com/), we classified the apps and games into 18 categories and seven categories, respectively. Then, we calculated individual’s frequency of using apps or games in different categories.
- We further examined temporal characteristics of created variables (see variables in (a) and (b)). During a 30-days observation, we calculated individual’s frequency of created variables within three time periods (morning: 6:00∼12:00; afternoon: 12:00∼18:00; evening: 18:00∼6:00), respectively.

We first calculated all behavioral features’ daily value for each user, then we calculated the average of each daily feature value, and finally we acquired average daily behavioral frequency for each user. Eventually, we extracted 105 types of smartphone variables (see Table 2).

**Table 2:** Details in smartphone usage behaviors variables.

Primary category	Detailed behaviors
App log	All App Activity logs Including 18 categories of App usage (except games): communication, media player, system, security, social, life, browser, inputting, beautify, reading, map, dictionary, news, money manage, office, photos, health, others Games: All games; Strategy game; Sport game; Intelligence game; Action game; Simulation game; Role playing game; Shooting game The top popular App usage: Tencent QQ; WeChat; RenRen; Sina microblog
GPS service	GPS service usage frequency Users’ daily range of movement according to GPS records
App package	Install/uninstall/replace/change/data clean/all operation frequency
SMS	Message number of all combinations of “sending/receiving messages,” “messages in the morning/afternoon/evening/all day,” “contact person is/isn’t in phone contacts,” a total of 24 features. The percentage of sending in all (received and send) messages
Call	Call number of all combinations of “making/receiving calls,” “calls in the morning/ afternoon/ evening/all day,” “contact person is/isn’t in phone contacts,” a total of 24 features. The percentage of outgoing in all (outgoing and incoming) calls The ratio between all SMS message numbers and all call numbers
Headsets	Headsets usage
Wallpaper	Wallpaper changing
Contacts	Contacts delete/add/all change frequency
Screen	Unlocking screen in the morning/ afternoon/ evening/all day
Charging	Phone charging

#### Social anxiety and loneliness

To examine differences in patterns of smartphone usage behaviors among users with different levels of social anxiety and loneliness, we divided participants into different groups (high-score, middle-score, and low-score group) based on their scores on social anxiety (means and standard deviations of scores: total 41.37 ± 8.711; men 42.54 ± 8.674; women 39.74 ± 8.578) and loneliness (means and standard deviations of scores: total 42.13 ± 9.270; men 42.45 ± 8.583; women 41.70 ± 10.220), respectively. To ensure balance of numbers in each group, in this study, we used extreme grouping method (Kelley, 1939; Feldt, 1961). Specifically, for each questionnaire (IAS or UCLA-LS), the top 27% of participants (34 participants) can be recognized as high-score group; while, the bottom 27% of participants (34 participants) can be identified as low-score group.

#### Statistics

We used SPSS 22.0 to conduct data analysis. In this study, only one variable “the average of daily ratio of outgoing call to all call” fitted normal distribution. We ran independent samples *T* test on this variable between high-score group and low-score group for IAS and UCLA-LS, respectively, and then we ran Wilcoxon-Mann-Whitney test on other variables for IAS and UCLA-LS, respectively.

## Results

### Smartphone usage behaviors and social anxiety

Firstly, we examined demographic variables (gender and age) of participants between high-score and low-score group for social anxiety. The result of Chi-square test on gender difference between high-score and low-score group showed no significance (χ^2^ = 2.946, *df* = 1, *p* = 0.086), and the result of independent samples *T* test on age differences between high-score and low-score group showed no significance (*t* = −1.911, *df* = 66, *p* = 0.066).

Secondly, we examined differences of smartphone variables between high-score and low-score group for social anxiety. The result of independent samples *T* test on variable “the average of daily ratio of outgoing call to all call” showed significance between high-score and low-score group (*t* = −0.848, *df* = 66, *p* = 0.022). For the Wilcoxon-Mann-Whitney test, significant results are shown in Table 3.

**Table 3:** The significant results of Wilcoxon-Mann-Whitney test between high-score and low-score group on IAS.

	High social anxiety score group			Low social anxiety score group			*Mann-Whitney U*	*Wilcoxon W*	*Z*	*P*
	Median	Min	Max	Median	Min	Max				
Total (incoming and outgoing) call	3.08	0.00	25.56	6.04	0.72	16.80	351.00	946.00	−2.78	0.005
Total call in the morning	1.03	0.00	16.71	1.57	0.06	3.93	399.00	994.00	−2.20	0.028
Total call in the afternoon	0.80	0.00	6.62	1.44	0.06	3.38	378.00	973.00	−2.45	0.014
Total call in the evening	1.51	0.00	5.82	2.43	0.41	11.43	329.00	924.00	−3.05	0.002
Incoming call	1.76	0.00	23.38	2.94	0.38	16.28	341.50	936.50	−2.90	0.004
Incoming call in the morning	0.53	0.00	21.21	0.88	0.13	3.08	360.00	955.00	−2.67	0.007
Incoming call in the afternoon	0.53	0.00	2.32	0.82	0.07	2.35	403.50	998.50	−2.14	0.032
Incoming call in the evening	0.47	0.00	3.00	1.30	0.09	12.23	311.00	906.00	−3.28	0.001
Incoming call from phone no. in contacts	0.86	0.00	23.09	1.98	0.19	4.85	293.00	888.00	−3.50	0.000
Incoming call from phone no. in contacts in the morning	0.23	0.00	21.12	0.55	0.03	1.93	321.00	916.00	−3.15	0.002
Incoming call from phone no. in contacts in the afternoon	0.25	0.00	2.23	0.52	0.00	1.33	370.50	965.50	−2.55	0.011
Incoming call from phone no. in contacts in the evening	0.29	0.00	2.14	0.86	0.06	3.83	282.50	877.50	−3.63	0.000
Outgoing call in the evening	0.82	0.00	4.15	1.48	0.00	3.44	409.00	1,004.00	−2.07	0.038
Total (receiving and sending) SMS in the afternoon	3.67	0.00	30.06	5.25	0.00	18.32	411.50	1,006.50	−2.04	0.041
Received SMS in the afternoon	2.07	0.00	22.17	3.63	0.00	9.90	400.50	995.50	−2.18	0.029
Received SMS from phone no. in contacts in the afternoon	0.58	0.00	11.71	1.53	0.00	4.55	391.50	986.50	−2.29	0.022
Health apps use	0.00	0.00	28.50	0.00	0.00	0.00	493.00	1,088.00	−2.30	0.021
Camera apps use	0.00	0.00	0.11	0.00	0.00	1.54	472.00	1,067.00	−2.11	0.035

### Smartphone usage behaviors and loneliness

Firstly, we examined demographic variables (gender and age) of participants between high-score and low-score group for loneliness. The result of the Chi-square test on gender difference between high-score and low-score group showed no significance (χ^2^ = 0.944, *df* = 1, *p* = 0.331), and the result of independent samples *T* test on age differences between high-score and low-score group showed no significance (*t* = 0.047, *df* = 66, *p* = 1.268).

Secondly, we ran the independent samples *T* test and Wilcoxon-Mann-Whitney test to examine differences in smartphone behaviors between high-score and low-score group. The result of the independent samples *T* test on variable “the average of daily ratio of outgoing call to all call” showed significance between high-score and low-score group (*t* = −0.497, *df* = 66, *p* = 0.028). For the Wilcoxon-Mann-Whitney test, significant results are shown in Table 4.

**Table 4:** The significant results of Wilcoxon-Mann-Whitney test between high-score and low-score group on UCLA-LS.

	High loneliness score group			Low loneliness score group			*Mann-Whitney U*	*Wilcoxon W*	*Z*	*P*
	Median	Min	Max	Median	Min	Max				
Total (incoming and outgoing) call	2.96	0.60	11.27	5.92	0.71	16.80	370.50	965.50	−2.55	0.011
Total call in the morning	0.94	0.00	9.64	1.43	0.06	5.16	394.00	989.00	−2.26	0.024
Total call in the afternoon	0.80	0.00	2.03	1.42	0.06	3.38	344.50	939.50	−2.86	0.004
Total call in the evening	1.24	0.00	5.26	2.40	0.29	11.43	362.00	957.00	−2.65	0.008
Incoming call	1.55	0.00	5.48	2.98	0.33	16.28	305.00	900.00	−3.35	0.001
Incoming call in the morning	0.52	0.00	2.17	0.89	0.00	4.11	321.50	916.50	−3.15	0.002
Incoming call in the afternoon	0.46	0.00	1.50	0.81	0.05	2.50	357.00	952.00	−2.71	0.007
Incoming call in the evening	0.50	0.00	3.38	1.42	0.05	12.23	314.00	909.00	−3.24	0.001
Incoming call from phone no. in contacts	0.91	0.00	4.87	1.78	0.03	9.55	373.50	968.50	−2.51	0.012
Incoming call from phone no. in contacts in the morning	0.30	0.00	1.91	0.44	0.00	4.05	407.50	1,002.50	−2.09	0.036
Incoming call from phone no. in contacts in the evening	0.31	0.00	2.35	0.84	0.00	3.83	354.50	949.50	−2.74	0.006
System apps use	104.88	0.00	1,597.95	16.81	0.00	245.90	314.00	909.00	−3.24	0.001
Beautify apps use	237.43	0.00	5,887.65	0.00	0.00	866.83	385.00	980.00	−2.52	0.012
Health apps use	0.00	0.00	29.50	0.00	0.00	0.00	510.00	1,105.00	−2.05	0.041
Browser apps use	10.42	0.00	1,151.40	4.07	0.00	89.12	416.00	1,011.00	−1.99	0.046
RenRen apps use	3.46	0.00	1,043.83	0.00	0.00	36.00	388.50	983.50	−2.60	0.009

## Discussion

This paper examines different types of smartphone usage behaviors and their relationships with social anxiety and loneliness by the Wilcoxon-Mann-Whitney test. Results showed that there exist differences in smartphone usage behaviors among users with different levels of social anxiety or loneliness.

For social anxiety, there were significant differences in 18 of 105 kinds of smartphone behaviors between high-score and low-score group. Participants in high-score group rarely make outgoing calls in the evening and receive less incoming calls at any time. Besides, they also seldom receive SMS in the afternoon. These results support some previous research, which suggests that individuals with social anxious often try to avoid initiating social interactions (Leary, 1983) and rarely receive social acceptance from others (Greca & Lopez, 1998). However, there are no significant differences in *sending SMS* between high-score and low-score groups, which is consistent with results of Reid’s research (Reid & Reid, 2007). In addition, social anxious individuals tend to use camera apps less frequently, which could indicate that they may have a small social network (Golder, 2008).

For loneliness, there were significant differences in 16 of 105 kinds of smartphone behaviors between high-score and low-score groups. Participants in high-score group receive less incoming calls at any time. Petersen et al. (2015) also found that loneliness is tightly associated with incoming but not outgoing calls. Besides, lonely individuals tend to use healthy apps more frequently, which can provide them online and ubiquitous access to health care services, and this finding confirms that lonely people prefer to use health care system (Geller et al., 1999).

In addition, in this study, some kinds of smartphone behaviors were not related with social anxiety and loneliness significantly. For example, smartphone behaviors indicating online social activeness (e.g. the usage frequency of social or communication apps) were not related with social anxiety and loneliness, which is consistent with conclusions of previous studies (Caplan, 2007). The other variables, such as frequency of phone charging, headset using, wallpaper changing, are not related to loneliness or social anxiety perhaps because these operations have nothing to do with loneliness or social anxiety.

This study has a few limitations. Firstly, since we only analyzed data acquired from 127 users, the results of this study might not be generalized to large population. Also participants were recruited from social media, the population is limited to those who use social media. Furthermore, the participants are very young and they might differ from other age groups. Secondly, some participants might have more than one smartphone or have other similar devices (e.g. pad, smart watch), which may lead to a limited collection of individual smartphone usage data. Thirdly, more temporal variables have not been investigated, such as the weekend and workday features, daily time series features. In future study, we plan to focus on larger samples, more kinds of smart devices (smart watch) and more smartphone behavior variables.

Despite these shortcomings, the results can be helpful to improve the early detection of social anxiety and loneliness. For one thing, this study found that smartphone usage behaviors can be used to identify targeted persons (those with higher levels of social anxiety and loneliness) timely. Results of this study support further investigation of the ways in building computational models for predicting individual’s scores on social anxiety and loneliness in real-time. For another thing, this study explored the way sociable persons use smartphones (e.g. the preference for using apps). Therefore, after identifying targeted persons, we can also provide them with some insightful suggestions (e.g. how to improve their social connections by smartphones).

## Conclusion

This study finds that relationships exist between smartphone usage behaviors and social anxiety and loneliness, which means we may be able to implement an early detection system of social anxiety and loneliness through smartphone usage behaviors. Besides, results of this study also provide useful suggestions to those who lack social interaction in daily lives.

## Supplemental Information

10.7717/peerj.2197/supp-1 Supplemental Information 1 All subjects’ social anxiety, loneliness scores, and their smartphone usage frequency.

## Additional Information and Declarations

### Data Deposition

The following information was supplied regarding data availability: The raw data has been supplied as Supplemental Dataset Files.
